# Predicting the Risk of Incident Type 2 Diabetes Mellitus in Chinese Elderly Using Machine Learning Techniques

**DOI:** 10.3390/jpm12060905

**Published:** 2022-05-31

**Authors:** Qing Liu, Miao Zhang, Yifeng He, Lei Zhang, Jingui Zou, Yaqiong Yan, Yan Guo

**Affiliations:** 1 Department of Epidemiology, School of Public Health, Wuhan University, Wuhan 430071, China; liuqing@whu.edu.cn (Q.L.); zhangmiao@whu.edu.cn (M.Z.); 2 School of Geodesy and Geomatics, Wuhan University, Wuhan 430079, China; heyifeng@whu.edu.cn (Y.H.); jgzou@sgg.whu.edu.cn (J.Z.); 3 School of Mathematics and Statistics, Wuhan University, Wuhan 430070, China; chris_lei@whu.edu.cn; 4 Wuhan Center for Disease Control and Prevention, Wuhan 430015, China; yanyyq@whcdc.org

**Keywords:** type 2 diabetes mellitus (T2DM), machine learning, prediction model, Chinese elderly

## Abstract

Early identification of individuals at high risk of diabetes is crucial for implementing early intervention strategies. However, algorithms specific to elderly Chinese adults are lacking. The aim of this study is to build effective prediction models based on machine learning (ML) for the risk of type 2 diabetes mellitus (T2DM) in Chinese elderly. A retrospective cohort study was conducted using the health screening data of adults older than 65 years in Wuhan, China from 2018 to 2020. With a strict data filtration, 127,031 records from the eligible participants were utilized. Overall, 8298 participants were diagnosed with incident T2DM during the 2-year follow-up (2019–2020). The dataset was randomly split into training set (*n* = 101,625) and test set (*n* = 25,406). We developed prediction models based on four ML algorithms: logistic regression (LR), decision tree (DT), random forest (RF), and extreme gradient boosting (XGBoost). Using LASSO regression, 21 prediction features were selected. The Random under-sampling (RUS) was applied to address the class imbalance, and the Shapley Additive Explanations (SHAP) was used to calculate and visualize feature importance. Model performance was evaluated by the area under the receiver operating characteristic curve (AUC), sensitivity, specificity, and accuracy. The XGBoost model achieved the best performance (AUC = 0.7805, sensitivity = 0.6452, specificity = 0.7577, accuracy = 0.7503). Fasting plasma glucose (FPG), education, exercise, gender, and waist circumference (WC) were the top five important predictors. This study showed that XGBoost model can be applied to screen individuals at high risk of T2DM in the early phrase, which has the strong potential for intelligent prevention and control of diabetes. The key features could also be useful for developing targeted diabetes prevention interventions.

## 1. Introduction

Diabetes mellitus (DM) is a chronic metabolic disease characterized by hyperglycemia, which can lead to serious complications such as chronic kidney disease, acute kidney injury, cardiovascular disease, ischemic heart disease, stroke, or even death [1]. Type 2 diabetes mellitus (T2DM) is the most common type of diabetes, accounting for around 90% of all diabetes cases. According to the report of the International Diabetes Federation (IDF) in 2021, about 537 million people worldwide are suffering from diabetes and the figure is projected to rise to 643 million by 2030 and 783 million by 2045 [2]. In China, it was estimated that there were 140.9 million adults living with diabetes, accounting for 25% of patients with diabetes worldwide [3]. The rising incidence of diabetes imposes a heavy burden on individual, health system, and the whole society [4,5].

T2DM is an irreversible but preventable disease [6]. Early diagnosis and effective screening of high-risk populations can prevent or delay the occurrence or development of T2DM and related complications [7]. Therefore, it is critical to establish an effective prediction model to assess individuals’ risk of T2DM, which would help the early identification of individuals at high risk of T2DM.

Machine learning (ML) is a subfield of artificial intelligence (AI) in computer science, which uses data-driven techniques to reveal patterns and predict behavior [8,9]. In recent years, machine learning techniques have been widely applied in the medical and health field, which have proven to be accurate and efficient in disease diagnosis, treatment, and prognosis [10,11]. There are many barriers to predict the risk of diabetes, because most of the medical data are nonlinear, nonnormal, correlation structured, and complex in nature [12]. Compared with traditional statistical methods, machine learning algorithms could learn the complex non-linear interactions between risk factors by minimizing errors between predicted and observed outcomes [13]. Predictive models based on machine learning algorithms can be useful in the identification of patients with diabetes and help discover hidden patterns in risk factors of diabetes that might be missed [14]. Numerous machine learning algorithms have been utilized for the prediction of T2DM, such as logistic regression (LR) [15], support vector machines (SVM) [16], artificial neural network (ANN) [17], k-nearest neighbors (KNN) [18], decision tree (DT) [19], random forest (RF) [20], and extreme gradient boosting (XGBoost) [21]. A recent meta-analysis confirmed the good discrimination ability of machine learning models to predict T2DM in community settings, suggesting that artificial neural network performed best, followed by logistic regression, decision trees, and random forests [22]. Xie et al. constructed several machine learning models for T2DM prediction using cross-sectional data of 138,146 participants in the United States, and the experimental results showed the neural network model gave the best model performance with the highest area under the receiver operating characteristic (AUC) value of 0.7949 [23]. The study of Katarya et al. based on Pima Indian diabetes dataset found that random forest performed the best with 0.84 accuracy and 0.83 AUC [24]. Adua et al. developed four machine learning classification algorithms (Naïve-Bayes (NB), KNN, SVM, and DT) to screen for T2DM in an African population, in Ghana, and concluded that NB algorithm performed best with the AUC of 0.87 [25]. A study in Luzhou, China utilized four ML algorithms to build prediction models of diabetes mellitus by using hospital physical examination data, and it revealed that random forest was the best performing model with the highest accuracy of 0.8084 [26]. A cross-sectional study in Urumqi, China based on the national physical examination data reported that XGBoost was the best classifier with AUC of 0.9680 [27]. Obviously, prior studies demonstrated different results in T2DM prediction even using the same machine learning algorithms [28]. Despite the extensive research on T2DM prediction, there were existing obstacles to applying prior prediction models, due to the disparity of study population, the difference of data sources, as well as the unsatisfactory power of those predictive models [29]. Thus, further studies including larger samples and elderly adults are still required to facilitate the research in this area.

This study aimed to build effective prediction models for the risk of incident T2DM among Chinese elderly adults based on four machine learning algorithms: logistic regression (LR), decision tree (DT), random forest (RF), and extreme gradient boosting (XGBoost). The purpose of this study was to provide evidence supporting the prevention and control of diabetes.

## 2. Materials and Methods

### 2.1. Study Design and Participants

**Figure 1:**
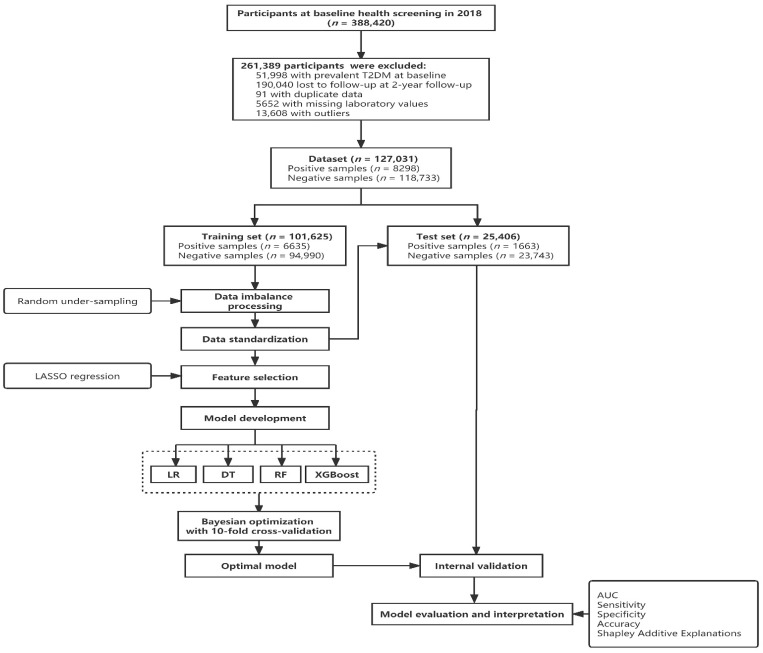
The study flow chart. LR, logistic regression; DT, decision tree; RF, random forest; XGBoost, extreme gradient boosting; LASSO, least absolute shrinkage and selection operator.

A retrospective cohort study was conducted using the health screening data of adults older than 65 years from 17 districts in Wuhan, China. The Wuhan Municipal Government would provide free physical examinations for the elderly aged 65 and above, which was regarded as a normalized and standardized project to benefit people. A total of 388,420 elderly people participated in the health screening in 2018. The protocol was approved by the Ethics Committee of Wuhan Center for Disease Control and Prevention (protocol code WHCDCIRB-K-2018023), and written informed consent was obtained from each participant. Baseline data were collected in 2018, and follow-up data were collected in 2019 and 2020. For longitudinal analysis of incident T2DM, excluding criteria of participants were: (1) Participants with prevalent T2DM at baseline (participants diagnosed by a fasting plasma glucose ≥7.0 mmol/L or with a self-reported previous diagnosis by health care professionals at baseline); (2) those who lost to follow-up; (3) those with duplicate data; (4) those with missing laboratory values; (5) those with outliers. After applying the exclusion criteria, a total of 127,031 participants were included in this study. The study flow chart is depicted in Figure 1.

### 2.2. Candidate Predictors

The health screening data were collected and recorded at the local community health service centers in Wuhan by well-trained research staff. It included three parts: a health status questionnaire, anthropometric measures, and laboratory measures. The questionnaire included age, gender, education, marital status, medical history (hypertension, myocardial infarction, coronary heart disease, angina pectoris, fatty liver), exercise, current smoking, current drinking. Anthropometric measures were conducted by trained medical staff using standardized procedures, including weight, height, waist circumference (WC), systolic blood pressure (SBP), and diastolic blood pressure (DBP). Body mass index (BMI) was calculated as weight (kg) divided by height squared (m^2^). Laboratory measures were performed at the central laboratory, including fasting plasma glucose (FPG), total cholesterol (TC), triglyceride (TG), high-density lipoprotein (HDL-C), low-density lipoprotein (LDL-C), alanine aminotransferase (ALT), aspartate transaminase (AST), total bilirubin (TBIL), serum creatinine (Scr), blood urea nitrogen (BUN), serum uric acid (SUA). The 27 candidate predictors from the health screening baseline data (Table 1) have been carefully selected based on the available variables in our dataset, clinical expertise, and prior literature evidence of their associations with T2DM [30,31,32].

### 2.3. Outcome

Incident type 2 diabetes mellitus (T2DM) was diagnosed if at least one of the following two criteria were satisfied according to the American Diabetes Association (ADA): (1) a self-reported diagnosis that was determined previously by a health care professional, or (2) fasting plasma glucose (FPG) ≥ 126 mg/dL (7.0 mmol/L) [33]. In this study, self-reported T2DM was defined by asking participants whether a health care professional had ever told that he/she was diagnosed with diabetes. Fasting blood samples were collected after at least 8 h of overnight fasting and were analyzed by trained research staff at the central laboratory. Fasting plasma glucose (FPG) levels were measured using the glucose oxidase procedure.

### 2.4. Machine Learning Algorithms

#### 2.4.1. Logistic Regression (LR)

LR is a classic classification algorithm that measures the relationship between a categorical dependent variable and one or more independent variables based on the sigmoid function [34]. This algorithm is a simple method for prediction which provides baseline accuracy scores to compare with other non-parametric machine learning models [14,35].

#### 2.4.2. Decision Tree (DT)

DT is a supervised learning technique used for a classification task. A decision tree is a class discrimination tree structure, with each internal node representing an attribute (or independent variable), each branch reflecting an outcome of the test, and each leaf node corresponding to a class label (or dependent variable) [11]. The purpose of DT is to generate a decision tree with strong generalization capability [36].

#### 2.4.3. Random Forest (RF)

RF is a typical ensemble learning algorithm that consists of multiple decision trees [37]. It can be applied to deal with regression and classification tasks. The algorithm is based on the idea of incorporating multiple decision tree classifiers to obtain the final classification result by majority voting and make accurate predictions [38]. RF can analyze complex interactions between characteristics, and is extremely adept at handling noisy and missing data [29].

#### 2.4.4. Extreme Gradient Boosting (XGBoost)

XGBoost is an advanced ensemble algorithm, which was proposed by Chen and Guestrin in 2016 [39]. It is a scalable machine learning technique for tree boosting that can combine a series of weak classifiers to construct a stronger classifier. This classifier is an optimized implementation of the gradient boosting decision tree (GBDT) and has the advantages of high training speed, excellent performance, and can deal with large-scale data.

### 2.5. Model Development

The dataset was randomly split into two parts: the training set accounted for 80% (*n* = 101,625) and the test set accounted for 20% (*n* = 25,406). Since the categories of the incident T2DM in the dataset were imbalanced, the Random under-sampling (RUS) was applied to the training set to resolve the effect of class imbalance. In order to standardize the input features, the data were normalized using the Python Sklearn library [40]. The training set was standardized to mean 0 and variance 1 using the StandardScaler function from the Sklearn preprocessing library in Python, and the test set was standardized using the mean and standard deviation of the training dataset. Least Absolute Shrinkage and Selection Operator (LASSO) regression was used for feature selection in the training set to construct the prediction models. LASSO is a regression model that penalizes the absolute sizes of the coefficients, resulting in the disappearance of some regression coefficients [41]. The candidates with non-zero coefficients are selected during the feature selection. We used LASSO regression with all candidate variables to screen the final input features for the prediction models.

We trained the logistic regression (LR), decision tree (DT), and random forest (RF) models implemented using the Python Sklearn package [42]. The extreme gradient boosting (XGBoost) was implemented using the Xgboost package [39]. The input variables were the 21 features selected by LASSO regression (Table 2). For the DT, RF, and XGBoost algorithms, Bayesian optimization with 10-fold cross-validation was performed on the training set to tune the hyperparameters. Bayesian optimization was proposed by Snoek et al. [43], which has demonstrated to outperform most global optimization algorithms on benchmark functions. It has become extremely popular for tuning hyperparameters in machine learning algorithms [44]. Bayesian optimization keeps track of the previous evaluation results of the objective function and uses them to create a surrogate model such as Gaussian process which was used to find out the most optimal hyperparameters [45]. After sufficient evaluations of the objective function until reaching maximum iterations, the surrogate function becomes an accurate model for the actual objective function and the set of hyperparameters selected is optimal [46]. After 500 iterations, we find the final optimal hyperparameters of DT, RF, and XGBoost. The best hyperparameters for DT were as followed: max_depth = 19, max_features = 7, min_samples_leaf = 55, min_samples_split = 10, min_weight_fraction_ leaf = 0.031159281996108103. The best hyperparameters for RF were as followed: max_depth = 68, max_features = 8, n_estimators = 80, min_samples_leaf = 5, min_samples_split = 69, min_weight_ fraction_ leaf = 0.0009215045821160297. The best hyperparameters for XGBoost were as followed: colsample_bytree = 0.6907621204231386, gamma = 0.6991315172625473, learning_rate = 0.093311071904797607, max_depth = 3, min_child_weight = 30, reg_alpha = 0.9430563747862351, reg_lambda = 0.7001632991135449, subsample = 0.5957497121054272.

### 2.6. Model Evaluation

The performances of the prediction models were evaluated on the test set using tuned hyperparameters. The area under receiver operating characteristic (AUC), sensitivity, specificity, and accuracy were used to evaluate the classification performance. Sensitivity indicates the proportion of positive sets being predicted correctly, and the specificity represents the proportion of negative sets being predicted correctly. Accuracy illustrates the correct prediction of both positive and negative sets. A receiver operating characteristic (ROC) curve was drawn with the true positive rate (sensitivity) as the ordinate and the false positive rate (1-specificity) as the abscissa, which indicates the overall performance of a binary classifier system. AUC was calculated from the ROC curve. The performance metrics were calculated as follows:

Sensitivity = TP/(TP + FN)
(1)

Specificity = TN/(FP + TN)
(2)

Accuracy = (TP + TN)/(TP + FP + TN + FN)
(3)

Here, TP, FN, FP, and TN represent true positive, false negative, false positive, and true negative, respectively.

### 2.7. Model Interpretation

For further model interpretation, the Shapley Additive Explanations (SHAP) was used. SHAP is a method proposed by Lundberg and Lee in 2017, which is widely used in the interpretation of various classification and regression models [47]. In this method, the features are ranked by their contribution to the model, and the relationship between features and the outcome can be visualized. The model would produce a predicted value for each sample, and the SHAP value represented the value allocated to each feature in the sample. Its absolute value reflects the influence of the feature, and its positive or negative reflects its positive or negative effect on the predicted risk of incident T2DM. When the SHAP value > 0, it indicated that the feature contributed to a higher risk of incident T2DM; On the contrary, when the SHAP value < 0, it indicated that the feature contributed to a lower risk of incident T2DM [48].

### 2.8. Statistical Analysis

Data analyses were performed using SAS version 9.4 and Python version 3.10. Baseline characteristics were summarized as means ± SD (standard deviation) for normally distributed continuous variables, as median and interquartile range (IQR) for non-normally distributed continuous variables, and as numbers and percentage for categorical variables. Students’ *t* test and Wilcoxon test were used to compare normal and non-normal continuous variables respectively and Chi-square tests or Fisher’s exact test were used to compare categorical variables between subgroups. The statistical significance level was set at *p*-value < 0.05 (two-sided). To implement the ML algorithms, we used the Python sklearn package [42] and the Xgboost package [39].

## 3. Results

### 3.1. Baseline Characteristics

Table 1 demonstrated the participants’ baseline characteristics. A total of 127,031 eligible participants were included in this study, which consisted of 8298 incident T2DM and 118,733 non-T2DM. The mean age of study participants was 71.94 ± 5.10 years old. The results showed that age, gender, education, marital status, hypertension, fatty liver, exercise, current smoking, BMI, WC, SBP, DBP, FPG, TC, TG, HDL-C, LDL-C, ALT, AST, TBIL, Scr, BUN, and SUA were all significantly associated with incident T2DM (*p* < 0.05).

### 3.2. Features Selected by LASSO Regression

Table 2 presented the results of the LASSO regression. Finally, 21 features were significantly associated with incident T2DM, including age, gender, education, marital status, hypertension, exercise, current smoking, current drinking, WC, SBP, FPG, TC, TG, HDL-C, LDL-C, ALT, AST, TBIL, Scr, BUN, and SUA.

### 3.3. Comparison of the Model Performance

**Figure 2:**
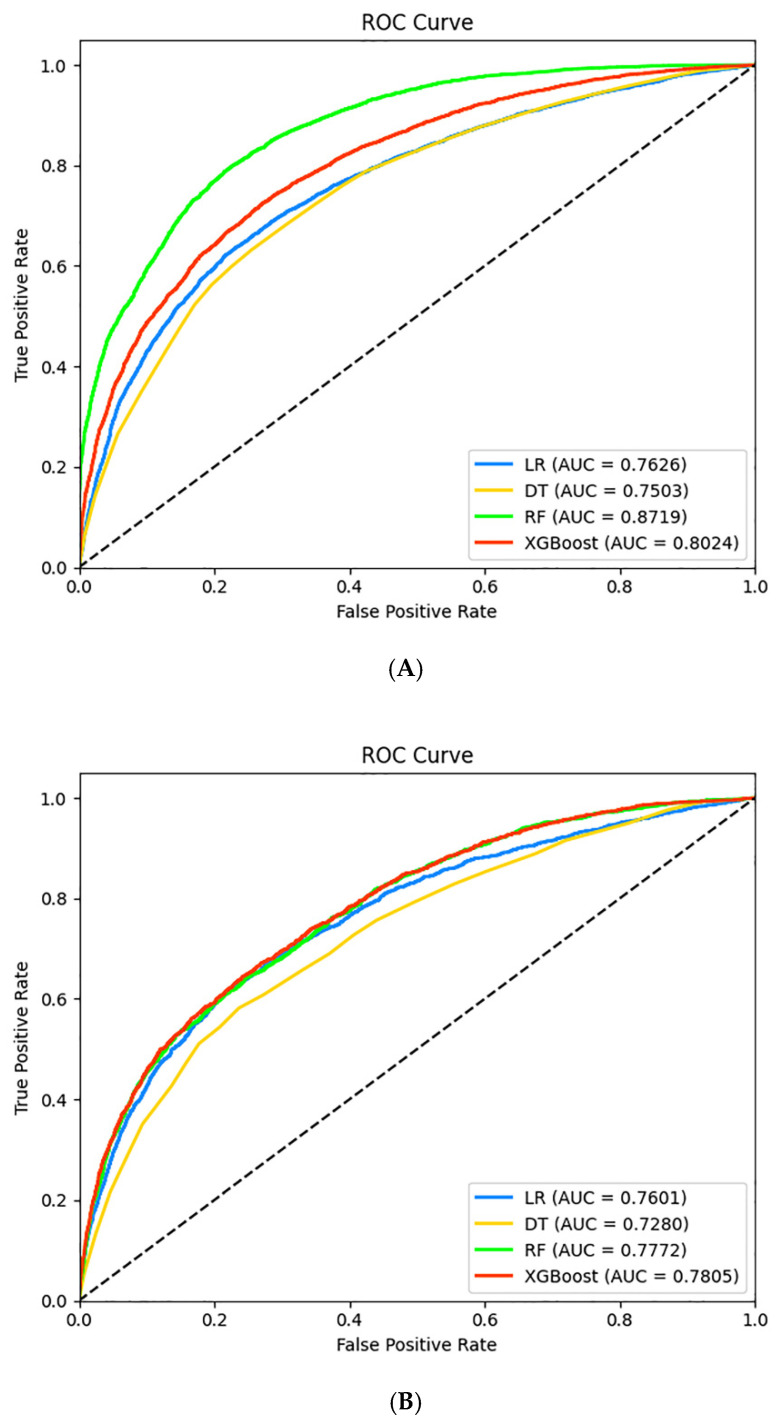
The receiver operating characteristics (ROC) curves of the four machine learning models on the training set (**A**) and test set (**B**).

**Figure 3:**
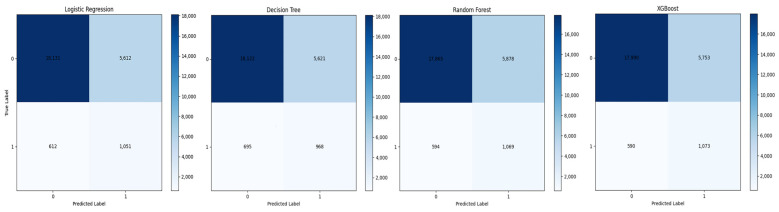
The confusion matrix of the four machine learning models.

Table 3 presented the results of performance of four machine learning models. The ROC curves on the training set and test set are shown in Figure 2. Overall, the XGBoost model performed best with the highest AUC value of 0.7805 on the test set, and the sensitivity, specificity, and accuracy were 0.6452, 0.7577, and 0.7503, respectively. The confusion matrix of the four machine learning models is presented in Figure 3.

### 3.4. Feature Importance

**Figure 4:**
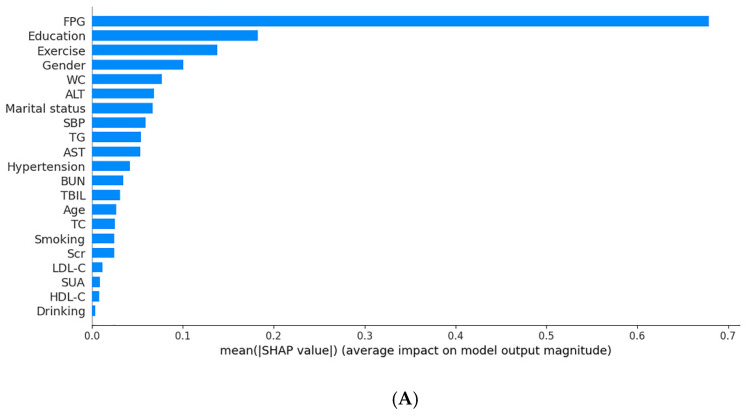

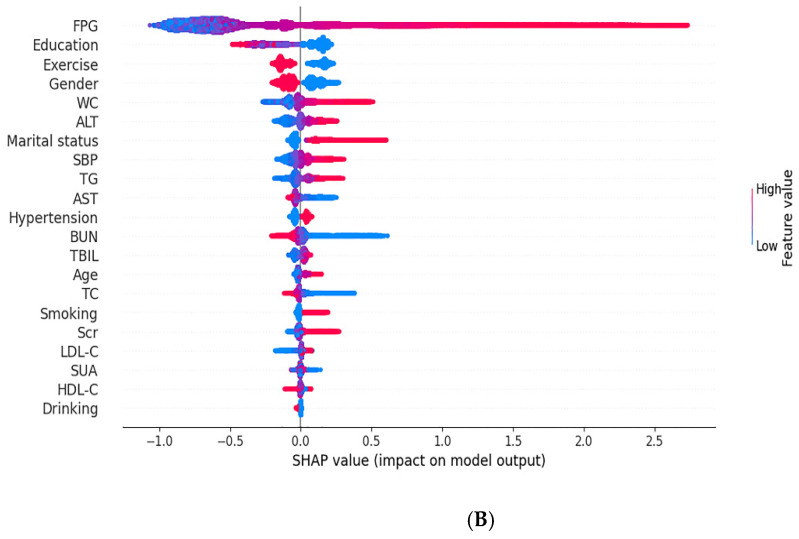
The interpretations for the XGBoost model. (**A**): The feature importance ranking by the SHAP value; (**B**): SHAP summary plot of the XGBoost model. Each dot represents a sample, with blue indicating a low feature value and red indicating a high feature value. The higher the SHAP value of a feature, the higher the risk of incident T2DM. Smoking was defined as current smoking; drinking was defined as current drinking.

In this study, XGBoost performed the best out of the four models. Figure 4 presented the contributions of the 21 features on the XGBoost model output ranked by the average absolute SHAP value. FPG, education, exercise, gender, and WC were the top five important features. The SHAP values of FPG, WC, ALT, marital status, SBP, TG, hypertension, TBIL, age, smoking, Scr, and LDL-C were greater than 0, which suggested that these features were significant risk factors for incident T2DM.

## 4. Discussion

In this retrospective study, we applied four machine learning algorithms to build prediction models for the risk of incident T2DM among Chinese elderly. It is found that the XGBoost model with 21 features demonstrated the best performance for predicting T2DM. This suggested that the prediction model derived in the present study could be applied to screen out individuals at high risk of T2DM, which could benefit the prevention and control of diabetes.

To date, the research of diabetes prediction models tended to focus on white populations [49,50,51,52], and Asian populations especially for the elderly have received relatively little attention. This study utilized a large longitudinal dataset obtained from Chinese elderly to establish prediction models for T2DM. The prediction results confirmed that the XGBoost model performed best with the highest AUC value of 0.7805 in predicting the probability that an individual develops T2DM. It was a good example of success for the XGBoost’s application in the research of diabetes risk prediction. This finding was consistent with earlier studies [14,21,27,53], which identified the good prediction power of the XGBoost model, with AUC values ranging from 0.8300 to 0.9680. Different from this study, a previous Korean population-based cohort study demonstrated that the ensemble models (e.g., stacking classifier) had better performance than the single models including XGBoost [54]. A rural cohort study in Henan province of China showed good predictive efficiency for the prediction models of T2DM, with AUC values ranging from 0.811 to 0.872 using laboratory data [55]. Compared with previous research, the AUC value in this study was relatively not satisfactory enough. A potential reason could be due to the differences of the study population and input features in the models, which could impact the predictive performance to some extent. Different from our study, the study population of prior studies [14,21,27,53] were middle-aged adults and fewer predictors were applied in the prediction of diabetes. To our knowledge, this was the first study that targeted the elderly population (≥65 years) in China to build predictive models for diabetes using machine learning techniques, which would have great implications for designing diabetes prevention focusing on the elderly. With the development of artificial intelligence, machine learning techniques have been widely applied in the medical field, especially for prediction models for diabetes [49,51,53,56,57,58]. It is worth noting that the advantages of machine learning models are well-documented empirically compared with traditional statistical methods, but its disadvantage is the lack of model interpretability [13]. XGBoost was often considered as a black box model, because it tends to have better accuracy for predictions compared with linear models while it loses the model interpretability at the same time [39]. Thus, we applied the Shapley Additive Explanations (SHAP) method developed by Lundberg and Lee [47] to better explain the contribution of each feature to the model. This is crucial for healthcare workers to get over the model interpretability barrier to apply predictive models in clinical practice.

Notably, the results of the feature importance analysis indicated the contribution of different feature to the model. These features such as FPG, education, exercise, gender, WC, etc., made substantial contributions to the prediction model. This was in accordance with the results observed in prior similar research [14,53,59]. Early identification of key risk factors had important implications for the risk assessment and prevention of diabetes. Our model results identified that FPG was the most significant predictor of T2DM. Individuals with higher blood glucose would have a greater likelihood of developing diabetes. An explanation for this was that hyperglycemia was correlated with insulin resistance [60]. As mentioned in the literature review, blood glucose was the main traditionally diabetes predictor and also widely used for diagnosis of diabetes [61]. This indicated that blood glucose control plays a key role in the prevention of T2DM, especially for the elderly.

As is shown in the present study, education and exercise showed negative associations with the risk of incident T2DM. Several studies have suggested that diabetes is associated with a low level of education [62,63,64,65,66]. A cohort study among American adults has confirmed that educational level was linked to the onset of diabetes [66]. Individuals with less than a high school educational level (hazard rate [HR] 1.58; 95% CI, 1.26–1.97) were more likely to develop diabetes. It is possible that people with higher education would have better health literacy, so they paid more attention to health management to prevent diabetes [65]. Prior studies have also noted the key role of exercise [67,68] and found that exercise intervention could decrease the risk of developing diabetes by 46% [68]. The China Da Qing Diabetes Prevention Study has identified the long-term effects of exercise interventions in reducing the incidence of T2DM [67]. It was shown that exercise intervention groups had a 49% decreased incidence of T2DM (hazard rate ratio [HRR], 0.51; 95% CI, 0.31–0.83) over the past two decades. There is need for implementing diabetes prevention programs, emphasizing the importance of regular exercise, and focusing particularly on lower educated populations. In our study, another interesting finding was that men were more likely to develop T2DM compared to women, which agreed with results from earlier studies [69,70]. Previous meta-analysis also demonstrated that gender was a dependent risk factor of T2DM in mainland China [71]. It found that the female gender (odds ratio [OR], 0.87, 95% CI, 0.78–0.97) was significantly negatively associated with the risk of T2DM. This could be explained by the fact that most risk factors (e.g., smoking and alcohol consumption, and physical inactivity) were more prevalent in men than women [72]. Therefore, more attention should be paid to men. As a measure of central/abdominal obesity, WC was also proved to be a strong predictor of T2DM. The significance of WC has been illustrated in other studies [17,73]. A 13-year prospective cohort study reported that a higher WC was linked to an increased risk of diabetes and the age-adjusted relative risks (RRs) across quintiles of WC were 1.0, 2.0, 2.7, 5.0, and 12.0, respectively [74]. Our findings further supported that the routine measurement of waist circumference would help clinical workers make preventive recommendations for individuals at high risk of diabetes.

Diabetes has become a major human health challenge and a global health burden because of its high morbidity and mortality rates [75,76]. The XGBoost prediction model established in this study showed promising performance. It had important public health implications, which could help clinicians screen out populations with a high risk of diabetes. The key features identified in this study not only captured each person’s socio-demographic variables, but also medical history, anthropometric and clinical laboratory variables, which could be effective for formulating and implementing targeted diabetes prevention strategies to reduce the disease burden.

Despite of the above encouraging findings, the current study has several limitations. First, only the participants who attended both the baseline survey and 2 -year follow-up were included in this study, which might potentially introduce a selection bias and limit the generalizability of the results. Second, some important risk factors of T2DM such as HbA1c, and insulin were not accounted for in the prediction models due to lack of relevant data. Third, some diabetes cases would be misclassified as non-T2DM because the oral glucose tolerance test (OGTT) was not included for the diagnosis of T2DM. However, the high cost and large sample size make it infeasible and difficult to perform oral glucose tolerance tests for all participants. Fourth, we only performed internal validation, and these prediction models need to be further validated in an external validation set in future work. Moreover, further work is warranted to consider auto encoder, to extract the type 2 diabetes mellitus (T2DM) features automatically, which can improve the classification efficiency of T2DM to some extent.

## 5. Conclusions

The current study developed four predictive models based on ML algorithms for the risk of incident T2DM in Chinese elderly. Our findings demonstrated that the XGBoost model achieved the best predictive performance for T2DM. Additionally, FPG, education, exercise, gender, and WC were the strongest predictors in the prediction model, which would benefit clinical practice in developing targeted diabetes prevention and control interventions.

## Figures and Tables

**Table 1:** Baseline characteristics of the participants.

Characteristics	Total (*n* = 127,031)	Incident T2DM		*p*-Value
		Yes (*n* = 8298)	No (*n* = 118,733)	
Age, mean (SD), years	71.94 (5.10)	72.39 (5.31)	71.91 (5.08)	<0.001
Gender, *n* (%)				<0.001
Men	56,774 (44.69)	4114 (7.25)	52,660 (92.75)	
Women	70,257 (55.31)	4184 (5.96)	66,073 (94.04)	
Education, *n* (%)				<0.001
Elementary school and below	75,828 (59.69)	5597 (7.38)	70,231 (92.62)	
Junior high school	28,298 (22.28)	1522 (5.38)	26,776 (94.62)	
Technical secondary school or high school	13,742 (10.82)	695 (5.06)	13,047 (94.94)	
Junior college and above	9163 (7.21)	484 (5.28)	8679 (94.72)	
Marital status, *n* (%)				<0.001
Married	98,131 (77.25)	6046 (6.16)	92,085 (93.84)	
Divorced	656 (0.52)	48 (7.32)	608 (92.68)	
Widowed	27,350 (21.53)	2082 (7.61)	25,268 (92.39)	
Single	894 (0.70)	122 (13.65)	772 (86.35)	
Hypertension, *n* (%)				<0.001
Yes	56,847 (44.75)	4347 (7.65)	52,500 (92.35)	
No	70,184 (55.25)	3951 (5.63)	66,233 (94.37)	
Myocardial infarction, *n* (%)				0.621
Yes	686 (0.54)	48 (7.00)	638 (93.00)	
No	126,345 (99.46)	8250 (6.53)	118,095 (93.47)	
Coronary heart disease, *n* (%)				0.413
Yes	7471 (5.88)	505 (6.76)	6966 (93.24)	
No	119,560 (94.12)	7793 (6.52)	111,767 (93.48)	
Angina pectoris, *n* (%)				0.711
Yes	506 (0.40)	31 (6.13)	475 (93.87)	
No	126,525 (99.60)	8267 (6.53)	118,258 (93.47)	
Fatty liver, *n* (%)				0.020
Yes	2279 (1.79)	176 (7.72)	2103 (92.28)	
No	124,752 (98.21)	8122 (6.51)	116,630 (93.49)	
Exercise, *n* (%)				<0.001
Yes	74,741 (58.84)	4323 (5.78)	70,418 (94.22)	
No	52,290 (41.16)	3975 (7.60)	48,315 (92.40)	
Current smoking, *n* (%)				<0.001
Yes	20,498 (16.14)	1515 (7.39)	18,983 (92.61)	
No	106,533 (83.86)	6783 (6.37)	99,750 (93.63)	
Current drinking, *n* (%)				0.908
Yes	21,429 (16.87)	1396 (6.51)	20,033 (93.49)	
No	105,602 (83.13)	6902 (6.54)	98,700 (93.46)	
BMI, mean (SD), kg/m^2^	23.70 (3.26)	24.47 (3.51)	23.65 (3.24)	<0.001
WC, mean (SD), cm	84.12 (9.16)	86.30 (9.62)	83.97 (9.10)	<0.001
SBP, mean (SD), mm Hg	137.12 (20.00)	140.63 (20.38)	136.87 (19.95)	<0.001
DBP, mean (SD), mm Hg	80.09 (11.20)	81.63 (11.42)	79.99 (11.18)	<0.001
FPG, mean (SD), mmol/L	5.12 (0.69)	5.71 (0.79)	5.08 (0.66)	<0.001
TC, median (IQR), mmol/L	4.81 (4.20–5.45)	4.84 (4.20–5.49)	4.81 (4.20–5.44)	0.034
TG, median (IQR), mmol/L	1.17 (0.85–1.63)	1.28 (0.90–1.79)	1.16 (0.85–1.62)	<0.001
HDL-C, median (IQR), mmol/L	1.36 (1.15–1.62)	1.32 (1.11–1.58)	1.37 (1.15–1.62)	<0.001
LDL-C, median (IQR), mmol/L	2.60 (2.08–3.17)	2.64 (2.11–3.24)	2.60 (2.07–3.16)	<0.001
ALT, median (IQR), U/L	16.00 (12.00–21.00)	17.00 (13.00–23.00)	16.00 (12.00–20.90)	<0.001
AST, median (IQR), U/L	21.50 (18.00–26.00)	22.00 (18.00–26.00)	21.50 (18.00–26.00)	0.004
TBIL, median (IQR), µmol/L	11.90 (9.17–15.30)	12.40 (9.50–15.90)	11.90 (9.10–15.30)	<0.001
Scr, mean (SD), µmol/L	76.82 (19.93)	79.21 (20.94)	76.66 (19.85)	<0.001
BUN, median (IQR), mmol/L	5.71 (4.76–6.82)	5.67 (4.70–6.80)	5.71 (4.77–6.83)	0.037
SUA, mean (SD), µmol/L	323.80 (91.90)	333.01 (94.31)	323.15 (91.70)	<0.001

SD, standard deviation; IQR: Q1–Q3 values; T2DM, type 2 diabetes mellitus; BMI, body mass index; WC, waist circumference; SBP, systolic blood pressure; DBP, diastolic blood pressure; FBG, fasting plasma glucose; TC, total cholesterol; TG, triglycerides; HDL-C, high-density lipoprotein cholesterol; LDL-C, low-density lipoprotein cholesterol; ALT, alanine aminotransferase; AST, aspartate transaminase; TBIL, total bilirubin; Scr, serum creatinine; BUN, blood urea nitrogen; SUA, serum uric acid.

**Table 2:** Least Absolute Shrinkage and Selection Operator (LASSO) regression coefficients.

Predictors	Coefficient
Age	0.012
Gender	−0.026
Education	−0.027
Marital status	0.023
Hypertension	0.010
Exercise	−0.035
Current smoking	0.017
Current drinking	−0.010
WC	0.033
SBP	0.014
FPG	0.219
TC	−0.022
TG	0.020
HDL-C	0.006
LDL-C	0.009
ALT	0.037
AST	−0.026
TBIL	0.006
Scr	0.004
BUN	−0.017
SUA	−0.002

**Table 3:** Comparison of performance of the four machine learning models.

Model	AUC	Sensitivity	Specificity	Accuracy
LR	0.7601	0.6320	0.7636	0.7550
DT	0.7280	0.5821	0.7633	0.7514
RF	0.7772	0.6428	0.7524	0.7453
XGBoost	0.7805	0.6452	0.7577	0.7503

## Data Availability

The data presented in this study are available from the corresponding author upon reasonable request.
